# Dietary and Behavioral Interventions for Diabetes: Current Evidence, Challenges, and Opportunities

**DOI:** 10.3390/nu17162604

**Published:** 2025-08-11

**Authors:** Noemí Lamonja-Vicente, David Lacasta Tintorer, Maria Palau-Antoja, Brenda Biaani León-Gómez, Pere Torán-Monserrat

**Affiliations:** Unitat de Suport a la Recerca Metropolitana Nord, Fundació Institut Universitari per a la Recerca a l’Atenció Primària de Salut Jordi Gol i Gurina (IDIAPJGol), 08303 Mataró, Spain; nlamonja@idiapjgol.info (N.L.-V.); dlacasta.mn.ics@gencat.cat (D.L.T.); mariapalauantoja@gmail.com (M.P.-A.); bleongo.mn.ics@gencat.cat (B.B.L.-G.)

Diabetes mellitus (DM) has emerged as one of the leading global health concerns due to its increasing prevalence and significant impact on healthcare systems [1,2]. Beyond pharmacological management, dietary and behavioral interventions have emerged as essential strategies for glycemic control, the prevention of diabetes-related complications, and the improvement of patients’ quality of life [3].

This Special Issue of *Nutrients*, entitled “Dietary and Behavioral Interventions for Diabetes”, provides a collection of critical studies evaluating the effectiveness and real-world applicability of said non-pharmacological interventions, compiling work on a range of methodologies, populations, and approaches, each highlighting the transformative potential of patient-centered strategies.

Bersch-Ferreira et al. conducted a multicenter randomized controlled clinical trial to assess the effectiveness of a multi-component dietary strategy on glycemic control (contribution 1). Although no differences were found between personalized dietary prescriptions and the multi-component approach, both interventions resulted in significant glycemic improvements. This underscores the importance of comprehensive nutritional planning for type 2 diabetes management, emphasizing the need to align dietary strategies with individual profiles and preferences in public health contexts.

Basiri and Cheskin’s study examined the effects of personalized nutrition therapy combined with continuous glucose monitoring (CGM) on body weight and composition in individuals with prediabetes (contribution 2). With a focus on conscious eating rather than explicit weight loss, it was found that personalized nutrition therapy led to significant reductions in weight and fat mass among overweight and obese prediabetic patients. These outcomes were further enhanced by the utilization of CGM devices, highlighting their potential as a behavioral change tool to allow patients to observe the immediate effects of dietary and physical activity changes on their glucose levels, and therefore enabling them to make informed decisions, reduce unnecessary dietary restrictions, and improve compliance.

Hybrid closed-loop insulin systems (HCLSs) have emerged as a primary approach for managing type 1 diabetes in children and adolescents, as they have demonstrated safety and efficacy in controlling glucose levels and insulin infusion. However, acute situations, such as attendance at summer camps, require adaptive changes in a short period of time to suit non-routine dietary and activity contexts for which these systems were not programmed. Olid-Cárdenas et al. investigated the effectiveness of HCLSs in pediatric diabetes camps, an environment where specific parameter configuration proved to be safe (contribution 3). These findings underscore the necessity of customizing technological interventions for the real-life results and outcomes of these systems, allowing adjustments for acute changes in schedules, activity, and diet.

Campbell et al. evaluated the acceptability of the Diabetes Remission Clinical Trial (DiRECT) within a population group with strong cultural emphases on food and shared eating in New Zealand through a qualitative study (contribution 4). The intervention’s success underscores the importance of individualized and culturally relevant behavioral support for effective weight loss and weight loss maintenance and reveals the key factors which influence the real-world implementation of lifestyle modification interventions, such as sociocultural context, family and community dynamics, and receiving caring professional support.

Gal et al. provided insights into dietary patterns and eating behaviors among type 2 diabetes patients, offering critical implications for tailored dietary education programs (contribution 5). As eating behavior influences dietary intake, understanding this relationship, as well as the importance of considering emotional, external, and other cofounding factors, could optimize diabetes management and allow for more individualized nutritional guidance to be achieved, improving not only glycemic control but also patients’ quality of life and overall health.

Finally, Garza et al. performed a systematic review and meta-analysis on aromatic herbs and spices typical of the Mediterranean Diet, highlighting their beneficial effects on glycemic profiles and offering new dietary tools for diabetes management (contribution 6). Although factors such as individual differences, dosage, or the combination of spices with changes in medication, physical activity, or lifestyle can affect the outcomes, aromatic herbs and spices improved fasting glucose, HbA1c, and insulin values overall, with ginger emerging as having a potential application in diabetes treatment, due to impacting all three parameters.

Each of these studies underscores the pivotal role of dietary and behavioral interventions, not merely as complementary strategies, but rather as central pillars of diabetes care. These approaches shed light on behavioral and metabolic mediators and demonstrate that there is significant potential to reduce reliance on pharmacological treatments, which often come with their own limitations and risks, thereby addressing the broader issue of medicalization in diabetes management.

From a public health perspective, these interventions offer cost-effective strategies, which are especially important in healthcare systems facing budget constraints and a high chronic disease burden. However, for their large-scale implementation, it is necessary to address structural, cultural, and behavioral barriers to ensure the inclusion of vulnerable populations and low-income countries, with issues such as adherence, equity, and healthcare professional training emerging as significant challenges requiring targeted solutions.

This Special Issue provides an integrated overview of non-pharmacological interventions in diabetes, highlighting the importance of dietary education, the promotion of physical activity, and equipping patients with self-management strategies. Moreover, it underscores the necessity for approaches that empower individuals with diabetes, enabling them to actively manage their health with interventions that adjust to their individual needs and their phenotypic diversity.

We sincerely thank all of the authors and reviewers who contributed to this timely and important collection of research. We hope that these contributions will inspire further research, innovative healthcare practices, and community empowerment in diabetes care.
